# Gradual differentiation uncoupled from cell cycle exit generates heterogeneity in the epidermal stem cell layer

**DOI:** 10.1038/s41556-022-01021-8

**Published:** 2022-11-10

**Authors:** Katie Cockburn, Karl Annusver, David G. Gonzalez, Smirthy Ganesan, Dennis P. May, Kailin R. Mesa, Kyogo Kawaguchi, Maria Kasper, Valentina Greco

**Affiliations:** 1 Department of Genetics, Yale School of Medicine, New Haven, CT USA; 2 Department of Cell and Molecular Biology, Karolinska Institutet, Stockholm, Sweden; 3 Nonequilibrium Physics of Living Matter RIKEN Habuki Research Team, RIKEN Center for Biosystems Dynamics Research, Kobe, Japan; 4 RIKEN Cluster for Pioneering Research, Kobe, Japan; 5 Universal Biology Institute, The University of Tokyo, Tokyo, Japan; 6 Departments of Cell Biology and Dermatology, Yale Stem Cell Center, Yale Cancer Center, Yale School of Medicine, New Haven, CT USA; 7 Present Address: Department of Biochemistry and Rosalind & Morris Goodman Cancer Institute, McGill University, Montreal, Quebec Canada

**Keywords:** Stem-cell differentiation, Multiphoton microscopy, Skin stem cells

## Abstract

Highly regenerative tissues continuously produce terminally differentiated cells to replace those that are lost. How they orchestrate the complex transition from undifferentiated stem cells towards post-mitotic, molecularly distinct and often spatially segregated differentiated populations is not well understood. In the adult skin epidermis, the stem cell compartment contains molecularly heterogeneous subpopulations^1–4^ whose relationship to the complete trajectory of differentiation remains unknown. Here we show that differentiation, from commitment to exit from the stem cell layer, is a multi-day process wherein cells transit through a continuum of transcriptional changes with upregulation of differentiation genes preceding downregulation of typical stemness genes. Differentiation-committed cells remain capable of dividing to produce daughter cells fated to further differentiate, demonstrating that differentiation is uncoupled from cell cycle exit. These cell divisions are not required as part of an obligate transit-amplifying programme but help to buffer the differentiating cell pool during heightened demand. Thus, instead of distinct contributions from multiple progenitors, a continuous gradual differentiation process fuels homeostatic epidermal turnover.

## Abstract (web summary)

Cockburn et al. report that epidermal differentiation is a multi-day process through which cells undergo a continuum of transcriptional alterations initiated independently of cell cycle exit.

## Main

During regeneration, the onset of stem cell differentiation and cell cycle exit are highly temporally correlated. However, many tissues contain intermediate cell types (often termed transit-amplifying cells) that have begun to differentiate but retain some proliferative capacity^5–7^. How such intermediate cell states arise and whether the overlap between proliferation and differentiation contributes to cellular maturation or tissue maintenance remains largely unclear.

**Extended Data Fig. 1:**
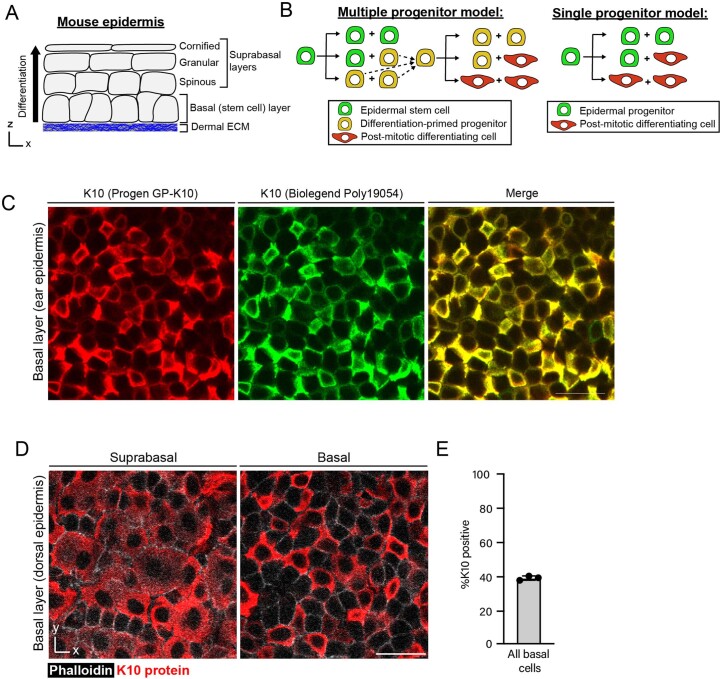
Characterization of Keratin 10 protein in basal cells. **(A)** Cartoon schematic of epidermal structure. Stem cells reside in an underlying basal layer (directly above dermal extracellular matrix, shown in blue), and differentiate upwards to contribute to the outer barrier layers of the skin. **(B)** Current models of epidermal homeostasis propose that the basal layer is composed of either multiple distinct stem/progenitor cells (one common model proposes slow cycling stem cells that give rise to a self-sustaining population of differentiation-primed progenitors; left cartoon) or a single type of epidermal progenitor (right cartoon). **(C)** Comparison of Keratin 10 whole mount immunostaining in ear epidermis using two independent antibodies (Progen GP-K10 in red; Biolegend Poly19054 in green). Scale bar=10 µm. **(D)** Representative whole mount staining of Keratin 10 (red) in suprabasal and basal cells from dorsal epidermis. Cell boundaries are visualized with phalloidin (white). Scale bar = 25 µm. **(E)** Quantification of percent dorsal basal cells that express Keratin 10 protein. Graph represents average from one independent immunostaining experiment using n=3 mice. Error bars are mean ±S.D. Source data

In the mammalian skin epidermis, proliferative basal layer cells differentiate and move upwards to replace barrier-forming cells that are continuously shed (Extended Data Fig. 1a). Lineage tracing of randomly labelled basal cells as well as label dilution studies support the existence of a single type of proliferating progenitor that generates dividing and differentiating cells with equal probability at the long-term, populational level^8–11^. In contrast, lineage tracing that reflects the more short term activity of distinct promoters such as Involucrin-CreERT2 (refs. ^1,2^), Tbx3-CreERT2 (ref. ^3^), Dlx-CreERT2 and Slc1a3-CreERT^4^ produces different distributions of clone numbers and sizes, indicating underlying differences in proliferative capacity or kinetics. Specifically, basal cells labelled by Involucrin-CreER have been suggested to represent a distinct and more differentiation-primed population of progenitors that contribute differentially to homeostasis versus wound repair^1^ (Extended Data Fig. 1b). How such progenitors might fit into the epidermal differentiation journey itself is not yet clear.

**Fig. 1:**
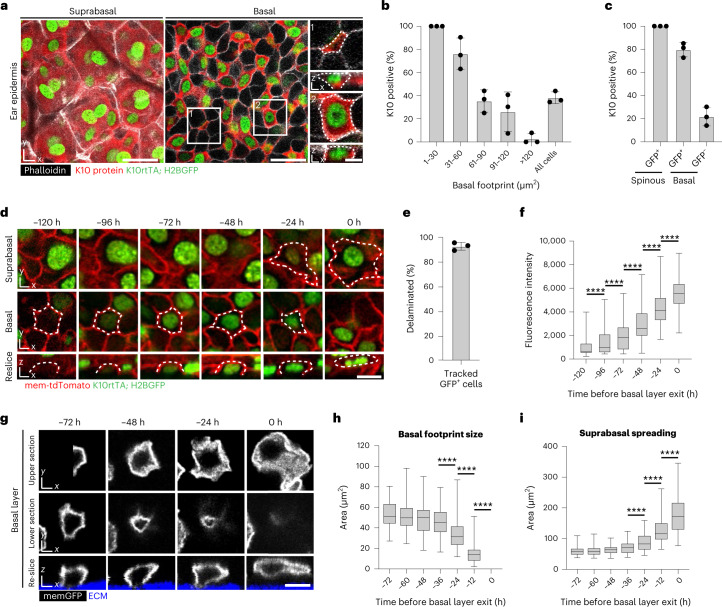
Epidermal stem cell differentiation occurs over multiple days. **a**, Whole-mount staining of K10 protein (red) and K10 reporter (K10rtTA; pTRE-H2BGFP) expression (green) in suprabasal and basal cells from ear epidermis. Cell boundaries are visualized with phalloidin (white). Insets show examples of K10-positive basal cells with very little (1) or average (2) amounts of ECM contact. Scale bars, 25 µm or 5 µm (insets). **b**, Percentage of basal cells with indicated levels of ECM contact that also express K10 protein. Graph represents average from one independent immunostaining experiment using *n* = 3 mice. **c**, Overlap between K10 protein expression and K10 reporter expression in basal and suprabasal cells. Graph represents average from one independent immunostaining experiment using *n* = 3 mice. **d**, Revisited basal cell as it induces K10 reporter expression and later exits the basal layer. Scale bar, 10 µm. **e**, Basal versus suprabasal position in basal cells scored as K10 reporter positive on day 0 and revisited for 5 subsequent days. Graph represents average of *n* = 3 imaged mice. **f**, K10 reporter expression levels in the days preceding basal layer exit. Graph represents 90 pooled cells from *n* = 3 imaged mice (see also tracks from individual cells in Supplementary Fig. 3). One-way ANOVA, *P* < 0.0001; Tukey’s HSD, *P* < 0.0001 (−120 h versus −96 h, −96 h versus 72 h, −72h versus −48 h, −24 h versus 0 h). **g**, Membrane GFP (K14CreER; mTmG) labelled basal cell revisited every 12 h in the days preceding delamination. Top row: *xy* section from the widest point in the upper half of the cell. Middle row: *xy* section directly above ECM (blue). Bottom row: lateral re-slice. Scale bar, 10 µm. **h**, Basal cell–ECM contact in the days preceding basal layer exit. Graph represents 72 pooled cells from *n* = 3 imaged mice. One-way ANOVA, *P* < 0.0001; 36 h versus 24 h, 24 h versus 12 h, and 12 h versus 0 h Tukey’s HSD, *P* < 0.0001. **i**, Suprabasal spreading, measured as the widest section in the upper half of each cell, in the days preceding delamination. Graph represents 68 pooled cells from *n* = 3 imaged mice. One-way ANOVA, *P* < 0.0001; 36 h versus 24 h, 24 h versus 12 h, and 12 h versus 0 h Tukey’s HSD, *P* < 0.0001. For **f**, **h** and **i**, box centres indicate median, boundaries represent 25th and 75th percentiles, and error bars represent maximum and minimum values. For bar graphs in **b**, **c** and **e**, error bars are mean ± standard deviation (s.d.). Source data

**Extended Data Fig. 2:**
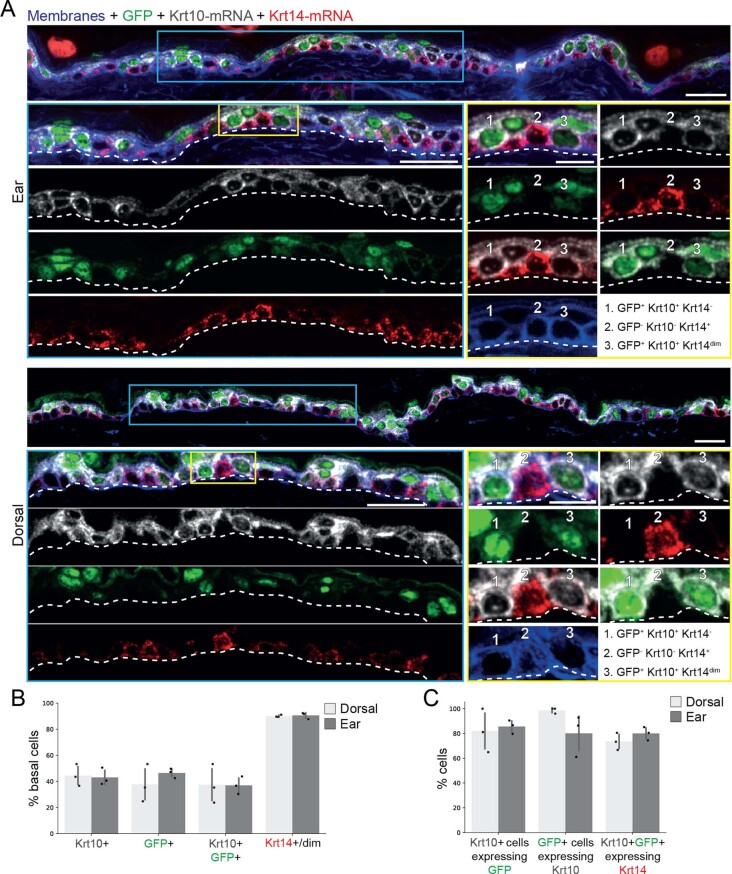
Characterization of *Keratin 10* transcripts in basal cells. **(A)** Tissue staining for GFP protein (*K10rtTA; pTRE-H2BGFP*) together with smFISH of *Krt10* and *Krt14* mRNA in ear and dorsal skin from the same genetic model used for *intra vital* imaging. Cell membranes were stained with WGA. Numbered cells in the zoom-ins show examples of cells with (1) *Krt10*-GFP protein and *Krt10* mRNA co-expression; (2) *Krt14* mRNA expression without *Krt10*-GFP nor *Krt10* mRNA expression; (3) *Krt10*-GFP protein, *Krt10* mRNA and *Krt14* mRNA (dim) co-expression. Scale bar = 25 μm (for overviews) or 10 μm (for zoom-ins). **(B-C)** Quantification of GFP, *Krt10* and *Krt14* expressing basal cells among all basal cells (**B**) and their respective co-expression within the same cells (**C**). n = 3 mice, error bars are mean ±S.D. Source data

To understand this journey in more detail, in this Letter, we focused on the long-standing observation that a subset of basal cells express the well-established differentiation marker keratin 10 (K10) (refs. ^12,13^). As these cells have typically been considered a post-mitotic population in the process of exiting the basal layer, neither their real-time behaviours nor their relevance to any of the aforementioned models of epidermal homeostasis have been closely examined. We first performed whole-mount immunostaining and single-molecule RNA fluorescence in situ hybridization (smRNA-FISH) in ear and dorsal skin (Fig. 1a and Extended Data Figs. 1c,d and 2). In total, 39–44% of basal cells stained positive for K10 protein or messenger RNA (mRNA), with the majority of these K10^+^ cells making a small area of contact (footprint) with the underlying extracellular matrix (ECM) as would be expected from delaminating basal cells (Fig. 1a,b and Extended Data Fig. 2a,b). However, we also observed K10^+^ cells with a typical basal-cell morphology including a normal-sized footprint (Fig. 1a,b and Extended Data Fig. 2a). These results indicate that, in contrast to previous models^14^, the differentiation process of basal epidermal cells may begin before a detectable change in cell morphology.

**Extended Data Fig. 3:**
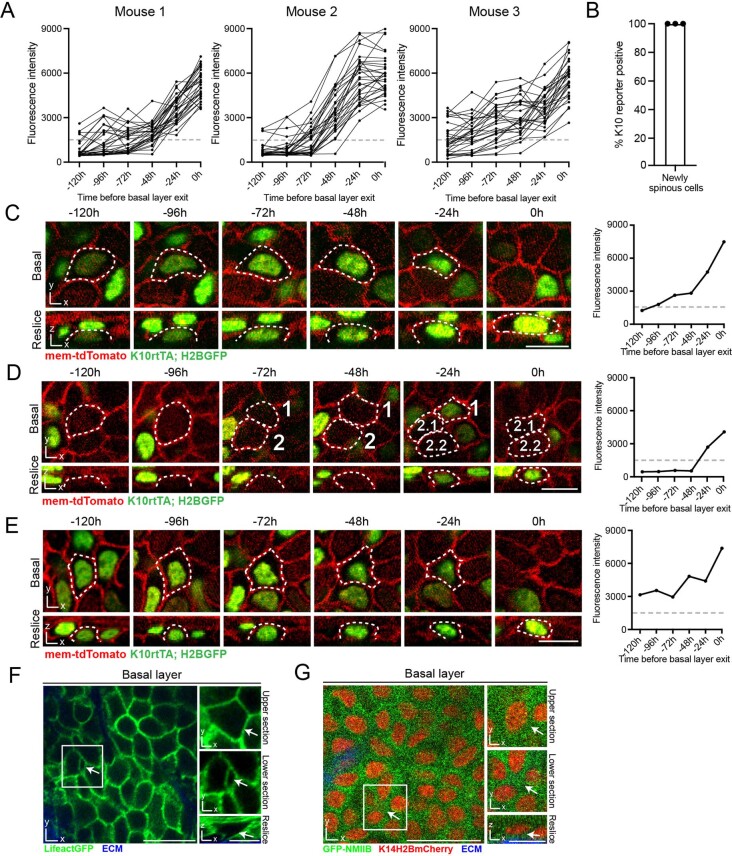
Characterization of Keratin 10 reporter expression and basal cell delamination. **(A)** Quantification of K10 reporter fluorescence in individual cells used for Fig. 1F. Dotted lines indicate the threshold used to score cells as reporter positive. **(B)** Percent tracked cells scored as K10 reporter positive at the timepoint prior to complete loss of ECM contact. N = 90 cells from 3 mice. Error bars are mean ±S.D. **(C, D and E)** Representative images and corresponding fluorescent quantification of three individual revisited basal cells as they induce K10 reporter expression and exit the basal layer. Each cell and fluorescent track were selected from the 90 cells from 3 independent mice shown in **(A)**. Scale bar = 10 µm. **(E)** Lifeact-GFP fluorescence (green) in the basal layer of ear epidermis. Insets show a cell in the process of delaminating (arrows). Top inset is an xy section from the upper half of the cell; middle inset is an xy section from directly above the ECM signal (blue); bottom inset is a lateral reslice. Image is representative of data from n = 3 mice. **(F)** GFP-tagged Non-muscle myosin IIB (*GFP-NMMIIB*) (green) in the basal layer of ear epidermis. K14H2B-mCherry signal (red) shows positions of epithelial nuclei. Insets show a cell in the process of delaminating (arrows). Top inset is an xy section from the upper half of the cell, containing the cell nucleus; middle inset is an xy section from directly above the ECM signal (blue); bottom inset is a lateral reslice. Image is representative of data from n = 3 mice. Scale bars for **(E)** and **(F)** = 20 µm or 10 µm (smaller insets). Source data

To understand the real-time behaviours of K10^+^ basal cells, we performed intra-vital imaging in mouse ear skin^10^ using a reporter system where the *Krt10* promoter drives H2BGFP fluorescence (*K10rtTA*; *pTRE-H2BGFP*)^15^ (Fig. 1a). This reporter labels 44% of basal cells, 80% of which co-express K10 protein (Fig. 1c) and/or *Krt10* mRNA (Extended Data Fig. 2c). By revisiting the same epidermal regions every 24 h (Fig. 1d), we found that the vast majority (93%) of K10-reporter-positive basal cells delaminate over the following 5 days (Fig. 1e), indicating that this population has largely committed to differentiate. We then focused on delaminating cells and tracked their H2BGFP signal in the days leading up to this event (Fig. 1f and Extended Data Fig. 3). Notably, K10 reporter expression always preceded delamination (Extended Data Fig. 3b), typically beginning 3–4 days before basal layer exit (Extended Data Fig. 3a,c–e). The corresponding morphological changes occurred surprisingly slowly over approximately 36 h (Fig. 1g–i). In contrast to the rapid, actomyosin-based extrusion events that have been described in embryonic epidermis and other systems^16–18^, basal cell delamination lacked obvious signs of ring-like actin or myosin accumulation (Extended Data Fig. 3f,g). These temporal observations demonstrate that adult basal cell differentiation, from commitment to the completion of delamination, is a gradual multi-day process.

**Fig. 2:**
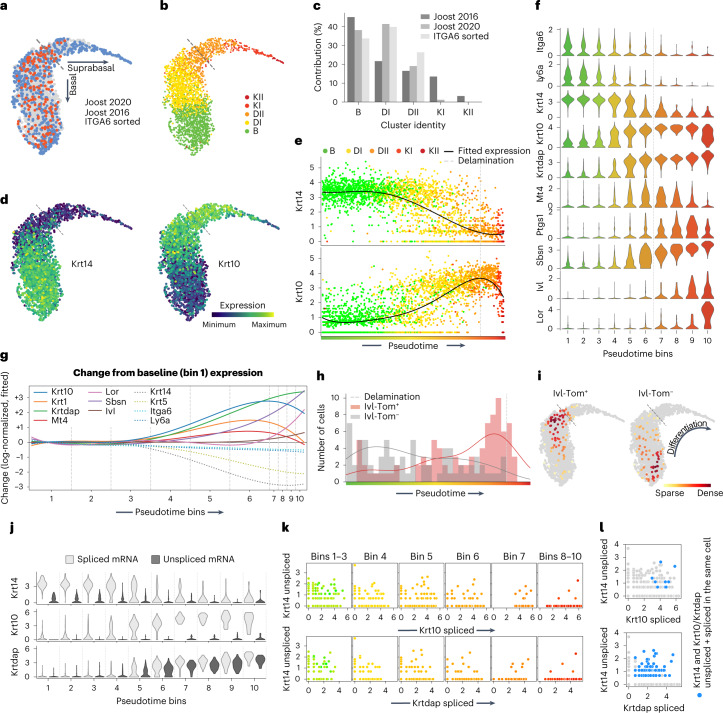
scRNA-seq trajectory analysis of epidermal stem cell and committed progenitor differentiation. **a**, UMAP of the combined epidermal datasets, showing the distributions for Joost 2020 (*n* = 5 mice), Joost 2016 (*n* = 19 mice) and ITGA6-sorted (*n* = 2 mice) cells. The dashed line indicates the assigned delamination point based on the location of 95% of ITGA6-sorted cells (Methods). **b**, Classification of cells in the combined dataset according to a kNN classifier based on the reference clusters from Joost 2016: basal (B), differentiating (DI and DII) and keratinizing (KI and KII). **c**, Bar plots showing the contribution of individual datasets to each cluster. **d**, *Krt14* and *Krt10* gene expression patterns overlaid on the combined UMAP. **e**, *Krt14* and *Krt10* expression (log-normalized) changes for individual cells, as well as fitted expression, ordered along pseudotime and coloured according to their differentiation state from basal (B) to differentiated (DI and DII) to keratinized (KI and KII) as defined in Joost 2016. **f**, Violin plots of differentiation-associated gene expression within cells grouped into ten pseudotime bins. **g**, Changes in fitted gene expression levels (log-normalized) of the genes shown in **f**, as compared with the baseline (average expression in bin 1). Solid and dashed coloured lines indicate genes that respectively increase or decrease their expression as compared with bin 1. The basal–suprabasal border is between bin 6 and bin 7. **h**, Distribution of *Ivl*-traced (Tom^+^) and non-traced (Tom^−^) cells along the pseudotime (histogram), together with estimation lines for cell density. **i**, Location of *Ivl*-traced (Tom^+^) and non-traced (Tom^−^) sorted cells on the combined UMAP. Cells are coloured according to the local density of visualized populations. **j**, Violin plots showing the spliced and unspliced mRNA expression levels (log) for *Krt14*, *Krt10* and *Krtdap* along the pseudotime bins. **k**, Scatter plots showing expression (log) of *Krt14*-unspliced mRNA and *Krt10*/*Krtdap*-spliced mRNA, separated into their respective pseudotime bins. **l**, Scatter plots showing the expression levels (log) of *Krt14*-unspliced and *Krt10*/*Krtdap*-spliced mRNA. Grey cells denote all the cells in the datasets, and blue cells co-express *Krt14* and *Krt10*/*Krtdap* spliced and unspliced mRNA. In **a**, **b**, **d**–**f**, **h** and **i**, dashed lines indicate the delamination point. In **d**–**f** and **j**–**l**, Expression is shown as log-normalized counts. Plots show integrated results of all biological replicates from all datasets combined (**a**–**g**), ITGA6-sorted cells (**h** and **i**) or Joost 2020 dataset cells (**j**–**l**).

**Extended Data Fig. 4:**
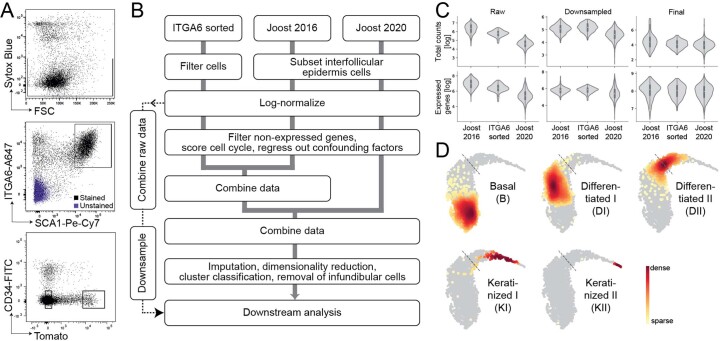
Merging scRNA-seq datasets. **(A)** Representative sorting strategy with gates set for live (upper panel), ITGA6 + and SCA1 + (middle panel), and CD34-negative cells according to their *Ivl*-Tomato expression (lower panel). **(B)** Flowchart describing the workflow of merging ITGA6-sorted, Joost 2016 and Joost 2020 dorsal skin datasets. Grey lines indicate which steps were performed individually for each dataset and when they were combined. Gene counts for expression analysis were combined from log-normalized counts. **(C)** Comparison of the total counts per cell and the number of genes expressed per cell for each dataset (Joost 2016 n = 19, ITGA6 sorted n = 2, Joost 2020 n = 5 mice). Only the final dataset genes (highly variable genes present in all datasets) were used for these comparisons. Note that an increase in the number of counts and expressed genes in the final dataset is an expected result of imputation. Y-axis values are shown as natural log. Inner boxplots show the median together with 1^st^ and 3^rd^ quartile, with whiskers denoting the minima and maxima of the distribution. **(D)** Locations of Joost 2016 cell clusters, overlaid on the combined UMAP, are colored according to local density of cells; all other cells are in grey. Dashed lines indicate the assigned basal-suprabasal border (delamination point). Source data

**Extended Data Fig. 5:**
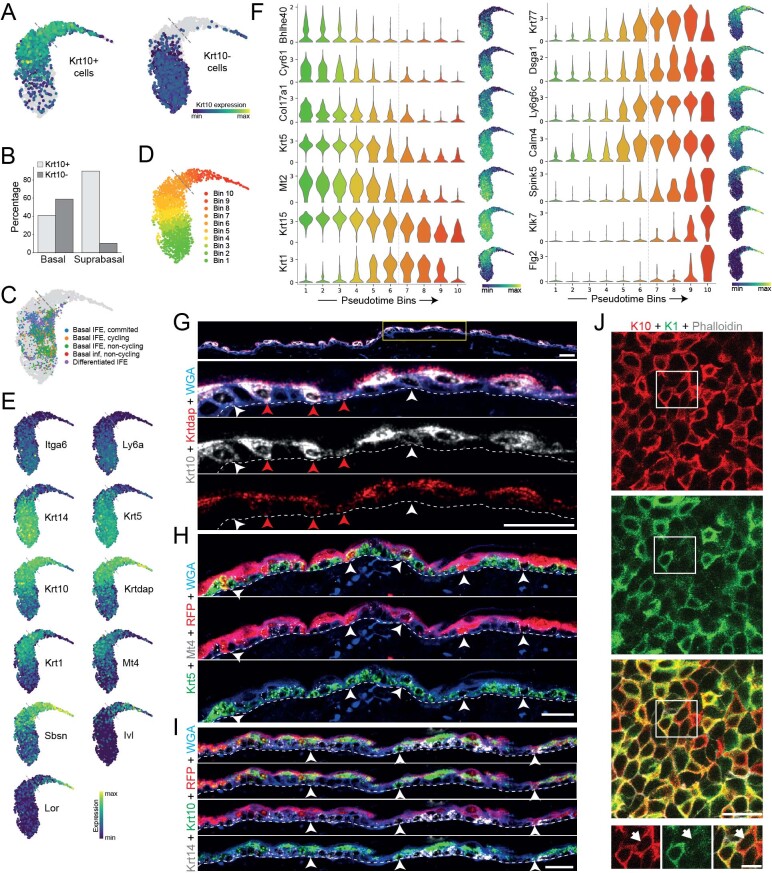
Characterization of the scRNA-seq datasets. **(A)** Classification of cells from all datasets as *Krt10*-positive or *Krt10*-negative (cutoff: 1.84 log-normalized and downsampled counts, see Methods). Cells are colored according to their *Krt10* expression levels. **(B)** Quantification of *Krt10*-positive and negative cells within the basal and suprabasal compartments (as defined by the delamination point). **(C)** Mapping of (Aragona et al., 2020) dorsal IFE dataset (colored) onto our combined UMAP (in grey). Colors indicate cluster annotations from Aragona dataset. **(D)** UMAP showing the grouping of cells into bins according to their location along the differentiation pseudotime. The basal-suprabasal border was set between bins 6 and 7 (Methods). **(E)** Expression patterns of genes shown in Fig. 2F, overlaid on the combined UMAP. **(F)** Violinplots grouped according to pseudotime bins (left panel) and combined UMAPs (right panel) showing characteristic gene expression changes during the differentiation process. Expression levels are shown as log-normalized expression. **(G)** smRNA-FISH validation of differentiation-associated gene expression in basal dorsal IFE cells. Arrowheads indicate basal cells with *Krt10*^+^ /*Krtdap*^+^ (red) or *Krt10*^+^ /*Krtdap*^−^ (white) expression. (**H-I**) smRNA-FISH and antibody-based stainings showing basal *Ivl*-traced cells (Tom^+^ stained via RFP) with *Krt5* and *Mt4* co-expression (arrowheads) (**H**), and with *Krt14* and *Krt10* co-expression (arrowheads) (**I**). (**G**-**I**) Cell membranes are stained with WGA (wheat germ agglutinin). Dashed lines indicate the basement membrane. Scale bars = 25 μm. (**A**, **C**, **D**, **E**, **F**) Dashed lines indicate the assigned basal-suprabasal border. (**J**) Representative whole mount staining showing K10 (red) and K1 (green) protein overlap in the basal layer of dorsal epidermis. Cell boundaries are visualized with phalloidin (white). Insets highlight an example of K10 positive, K1 low cells (arrowhead). Scale bar = 25 µm (large field of view) or 10 µm (inset). Images in (**G**-**J**) are representative of staining from n = 3 mice. (**A-F**) Plots show integrated results of all biological replicates from all datasets combined (Joost 2016 n = 19, ITGA6-sorted n = 2, Joost 2020 n = 5 mice) with exception of (**C**) which includes additional integration of Aragona dataset.

We next aimed to understand how K10 expression relates to the global transcriptional changes associated with basal cell differentiation and to other previously described differentiation-primed progenitor populations^1,2^. Our previous single-cell transcriptome-based reconstruction of the epidermal differentiation trajectory grouped cells according to their individual gene expression from basal (*Krt14*^high^) to mature (*Krt10*^high^) to terminally differentiated (*Lor*^high^) cell states^19^. To define the basal-suprabasal border on this trajectory, we generated single-cell transcriptomes of fluorescence-activated cell sorting (FACS)-isolated basal cells (Live/ITGA6^+^/SCA1^+^/CD34^−^; Extended Data Fig. 4a). We then merged these transcriptomes with two published datasets encompassing both basal and suprabasal cells (Extended Data Fig. 4b–d and Methods)^19,20^, which allowed us to assign the basal-suprabasal border according to the sorted basal (ITGA6^+^) cells (Fig. 2a and Methods). Notably, the previously defined intermediate *Krt10*^dim^/*Ptgs1*^dim^/*Mt4*^+^ cell group (Differentiated I) and part of the mature *Krt10*^high^/*Ptgs1*^high^ group (Differentiated II) (ref. ^19^), are basal-layer cells (Fig. 2b,c). This is exemplified by ~40% of basal cells already expressing *Krt10* transcripts (Extended Data Figs. 2a,b and 5a,b), as well as other basal and differentiation transcripts in the same cells (Fig. 2d,e and Extended Data Fig. 5e–i), consistent with recent studies in oral^21^ and skin epithelia (Extended Data Fig. 5c)^22^.

**Extended Data Fig. 6:**
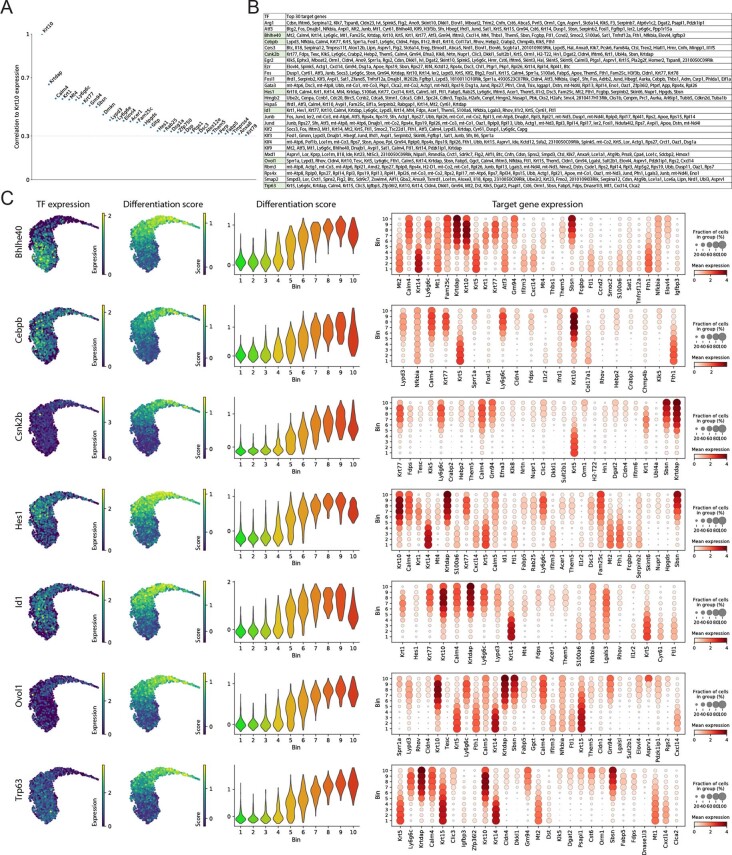
Regulatory network analysis with Scenic. **(A)** Genes correlated with *Krt10* expression in the early stages of differentiation (bins 2, 3 and 4). Shown are all genes with a pearson correlation > 0.3. **(B)** Identified transcription factors that target at least one of the *Krt10*-correlated genes and their corresponding targets (top 30, importance >10). Transcription factors highlighted in green were selected for plotting in (C). **(C)** Plots showing expression patterns of selected transcription factors and their putative target and/or co-regulated genes from Scenic analysis. Transcription factors were selected by their target gene expression patterns and literature search. Panels show the expression of the transcription factor overlaid on UMAP (left), differentiation-related target gene expression score overlaid on UMAP (middle-left), violinplot of differentiation-related target gene expression score grouped according to differentiation bins (middle-right), and dotplot of top 30 (or all for *Cebpb*) target gene expression patterns (right). Dot plot genes were ordered according to their importance as a target gene for the transcription factor. Differentiation-related target genes were defined as genes that were not identified as differentially expressed in bin 1. (**A-C**) plots show integrated results from all biological replicates from all datasets (Joost 2016 n = 19, ITGA6-sorted n = 2, Joost 2020 n = 5 mice).

To examine early transcriptional changes signifying the onset of differentiation more closely, we grouped the cells along the trajectory into ten differentiation bins (Fig. 2f, Extended Data Fig. 5d–f and Methods), revealing *Krt10* as the first upregulated differentiation marker (bin 3), followed by *Krtdap* and *Krt1* (bin 4), *Mt4* and *Sbsn* (bin 5) and *Ivl* and *Lor* (bin 6) (Fig. 2f,g and Extended Data Fig. 5e–i). Consistently, immunostaining revealed a small subset of K10^+^ basal cells with very low levels of K1, but not vice versa (Extended Data Fig. 5j). Basal marker-gene expression starts to decrease in bin 3–4 (*Itga6* and *Ly6a*), with the decrease of *Krt14* being most pronounced (Fig. 2f,g and Extended Data Fig. 5e,f). Thus, the earliest molecular changes associated with differentiation start in bin 3, marked by an increase of *Krt10* expression in cells displaying otherwise typical characteristics of stem cells (*Krt14*^high^, large ECM footprint) (Extended Data Fig. 5g–i). In addition, we observed gene-expression patterns associated with known as well as less explored transcription factors (TFs; *Id1*, *Hes1*, *Trp63*, *Ovol1* and *Csnk2b*), all leading to a gradual onset of differentiation-associated gene expression (bins 2–4; Extended Data Fig. 6).

Our results indicate that, instead of discrete intermediate cell states, basal cells differentiate through a series of progressive transcriptional changes, raising questions about how previously proposed differentiation-primed progenitors (that is, Involucrin-CreER traced cells^1,2^) fit within this continuum. Thus, we acquired our ITGA6-sorted dataset from Involucrin-CreER traced (committed; Tom^+^) and non-traced cells (non-committed; Tom^−^) (Extended Data Fig. 4a). The majority of Tom^+^ basal cells indeed mapped with the *Krt10*^+^/*Mt4*^+^ cells and the majority of Tom^−^ cells mapped with basal *Krt14*^+^ cells (Fig. 2h,i and Extended Data Fig. 5h,i), suggesting that the Involucrin-traced cell population largely represents committed cells but not necessarily a discrete progenitor cell state.

We next sought to investigate whether upregulation of differentiation genes precedes the downregulation of stem cell signature genes or vice versa. As splicing typically occurs co-transcriptionally 5–15 min after crossing an exon–intron junction^23,24^ we analysed spliced and unspliced *Krt10* and *Krt14* mRNA fragments during the defined differentiation process (Fig. 2j). Focusing on differentiation pseudotime stages that had both high *Krt10* and *Krt14* expression (bins 4–6), we observed that *Krt14* is still actively transcribed (unspliced mRNAs) in cells with high expression of mature (spliced) *Krt10* mRNA (Fig. 2j,k). Moreover, active transcription (unspliced) of both *Krt14* and *Krt10* mRNA can occur within the same cells (Fig. 2l). Additionally, *Krt14* transcription can remain active even in differentiating, *Krtdap*-expressing cells (Fig. 2j–l). In sum, this fine-tuned differentiation trajectory appoints *Krt10* expression at the molecular onset of a continuum of transcriptional changes associated with epidermal differentiation.

**Fig. 3:**
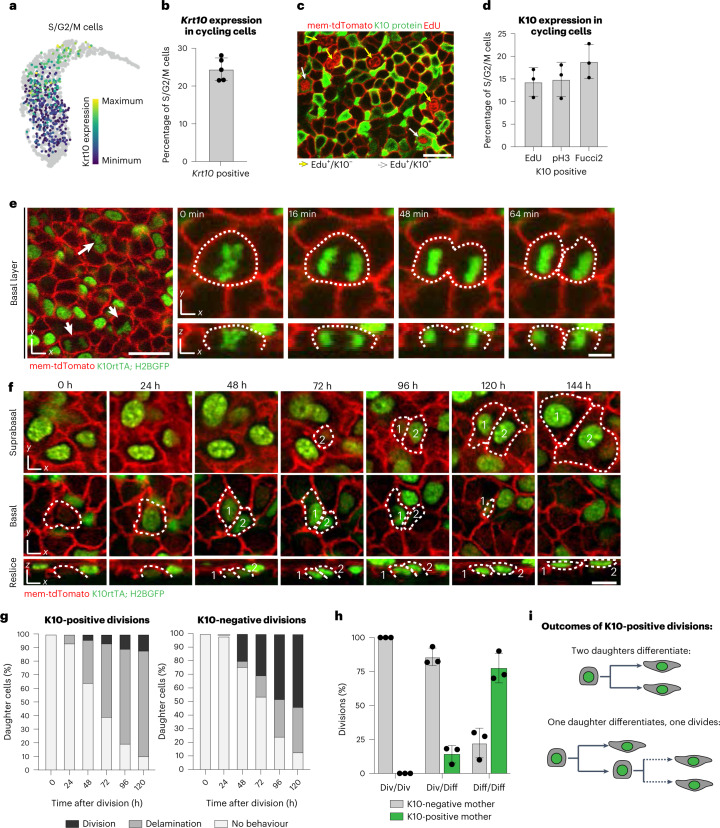
Differentiation-committed cells proliferate during homeostasis. **a**, UMAP showing *Krt10* gene expression levels in all cycling (S/G2/M) epidermal cells from the Joost 2020 dataset. **b**, Quantification of *Krt10*-positive cells (cut-off 1.84, Methods) within the proliferative cell population (S/G2/M) from the Joost 2020 dataset. Graph represents average of independent experiments from *n* = 5 mice. **c**, Representative whole-mount staining of EdU incorporation (red nuclei), K10 (green) and mem-tdTomato (red membrane) showing both EdU-positive, K10-negative (yellow arrows) and EdU-positive, K10-positive (white arrows) basal cells. Scale bar, 20 µm. **d**, Proportion of actively cycling cells (indicated by EdU, pH3 or Fucci2 mVenus-hGem positivity) that also express K10 protein. Graph represents average from one independent immunostaining experiment using *n* = 3 mice for each proliferative marker. Error bars indicate s.d. **e**, Single timepoints (left) or stills from timelapse imaging (right) show K10-reporter-positive (green) mitotic figures, indicated by white arrows (left) or dotted lines (right). Timelapse imaging captures K10 reporter positive divisions generating two basal daughter cells. Membrane is visualized with mem-tdTomato (red). Scale bars, 20 µm (large field of view) or 5 µm (timelapse stills). **f**, Representative images of a revisited basal cell as it induces K10 reporter expression and divides to produce two daughter cells (numbered 1 and 2) that exit the basal layer. Scale bar, 10 µm. **g**, Cumulative daughter fates in the first 5 days following K10-reporter-positive and K10-reporter-negative divisions. *N* = 76 cells from three mice (K10-reporter-positive divisions) and 228 cells from three mice (K10-reporter-negative divisions). **h**, Quantification of division modes as asymmetric (div/diff), symmetric with both daughters differentiating (diff/diff), or symmetric with both daughters dividing (div/div) from all division events where the subsequent behaviour of both daughters could be resolved in later revisits. Graph represents average of *n* = 3 imaged mice. **i**, Schematic of daughter cell fates after K10-reporter-positive divisions. For bar graphs in **b**, **d** and **h**, error bars are mean ± s.d. Source data

**Extended Data Fig. 7:**
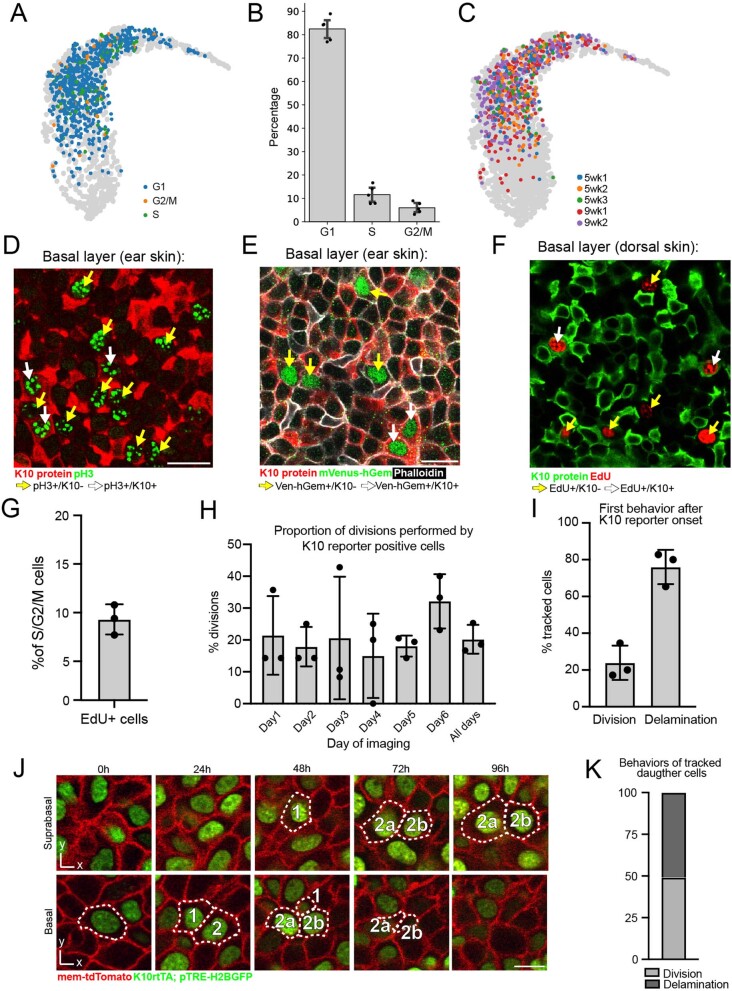
Characterization of Keratin 10-positive cell divisions. **(A)** UMAP representation of cell cycle phases of Joost 2020 cells that were classified as *Krt10*^+^. **(B)** Quantification of the proportion of S and G2/M phase cells within the *Krt10*^+^ cell population shown in (**A**); graph represents average of independent experiments from n = 5 mice. **(C)** UMAP representation of *Krt10*-expressing cells from the 5 biological replicates from Joost 2020, overlaid on the combined UMAP (in gray). **(D)** Whole mount staining of pH3 (green) and K10 (red) in the basal layer of ear skin, showing both pH3-positive, K10 negative (yellow arrows) and pH3-positive, K10-positive (white arrows) cells. Scale bar = 20 µm. **(E)** Whole mount staining of mVenus-hGem signal from Fucci2 reporter mice (green), K10 (red) and phalloidin (white) in the basal layer of ear skin, showing both mVenus-hGem-positive, K10 negative (yellow arrows) and mVenus-hGem-positive, K10-positive (white arrows) cells. Scale bar=20 µm. **(F)** Whole mount staining of EdU (red) and K10 (green) in the basal layer of dorsal skin, showing both EdU-positive, K10 negative (yellow arrows) and EdU-positive, K10-positive (white arrows) cells. Scale bar=20 µm. For (D-F), staining is representative of n = 3 mice. **(G)** Proportion of EdU positive cells that also express K10 protein in dorsal skin. Graph represents average from one independent immunostaining experiment using n=3 mice. **(H)** Proportion of divisions performed by K10 reporter positive cells in revisited basal regions. Graph represents average of n=3 imaged mice. **(I)** Proportion of K10 reporter positive cells performing either division or delamination as their first behavior. Analysis was limited to cells that could be seen inducing K10 reporter expression and then performing a subsequent behavior (either division or delamination) within the 6-day revisit window. Graph represents average of n=3 imaged mice. **(J)** Revisited K10-positive basal cell as it divides to produce one delaminating daughter cell (number 1) and a second daughter cell (number 2) that goes through another round of cell division before exiting the basal layer (numbers 2a and 2b). Scale bar = 10 µm. Image representative of data from n = 3 mice. **(K)** Fates of all daughter cells from Fig. 3G whose first behavior could be resolved within 5 days of birth. N = 266 cells from 3 mice. For (**B**), (**G**), (H) and (**I**), error bars are mean ±S.D. Source data

Further, analysis of the cell cycle stages revealed that approximately 15% of *Krt10*-expressing cells are actively proliferating (Extended Data Fig. 7a–c). *Krt10*^+^ cells account for ~24% of the S/G2/M phase population (Fig. 3a,b), while K10 protein is detectable in 9–19% of these cells (Fig. 3c,d and Extended Data Fig. 7d–g). Through detailed timelapse imaging, we observed that K10^+^ cells undergo mitosis parallel to the basement membrane and produce daughter cells that are fully integrated within the basal layer and retain reporter expression (Fig. 3e and Supplementary Video 1). Moreover, 20% of all divisions were performed by reporter-positive cells (Extended Data Fig. 7h). Conversely, 22% of cells that induced K10 reporter expression underwent division as a subsequent behaviour (Extended Data Fig. 7i). In the 5 days following their birth, most daughter cells (78%) from K10^+^ divisions had delaminated, completing the differentiation trajectory begun by their mother cell (Fig. 3f,g). More rarely (12%), daughter cells underwent an additional round of division. In cases when daughters from these subsequent divisions could be further tracked, we often witnessed them also delaminating (Extended Data Fig. 7j). These results contrast with K10^−^ divisions, where 33% of daughters delaminated and 53% divided in the same time frame (Fig. 3g). Overall, of the 266 daughter cells whose subsequent behaviour after birth could be resolved within 5 days, 51% delaminated and 49% divided (Extended Data Fig. 7k), indicating that the population tracked in our short-term revisits is representative of the homeostatic basal population.

We next focused on basal K10-reporter-positive divisions to understand how they contribute to asymmetric or symmetric fate outcomes (Extended Data Fig. 1b, right)^1,8^. Among divisions where the behaviour of both daughter cells could be resolved in subsequent days of imaging, K10^+^ mother cells produced 76% of all divisions with two symmetrically differentiating daughters, 18% of all asymmetric divisions and no divisions with symmetrically dividing daughters (Fig. 3h,i). This preponderance of symmetric, differentiation-fated divisions contrasts with the largely asymmetric, self-renewing mode of division that has been proposed to characterize differentiation-primed progenitors in other models^1,2^. Together these results indicate that, although K10 reporter expression signifies commitment to eventually delaminate, differentiating cells remain capable of dividing to generate short-term lineages of basal daughter cells fated to delaminate.

**Fig. 4:**
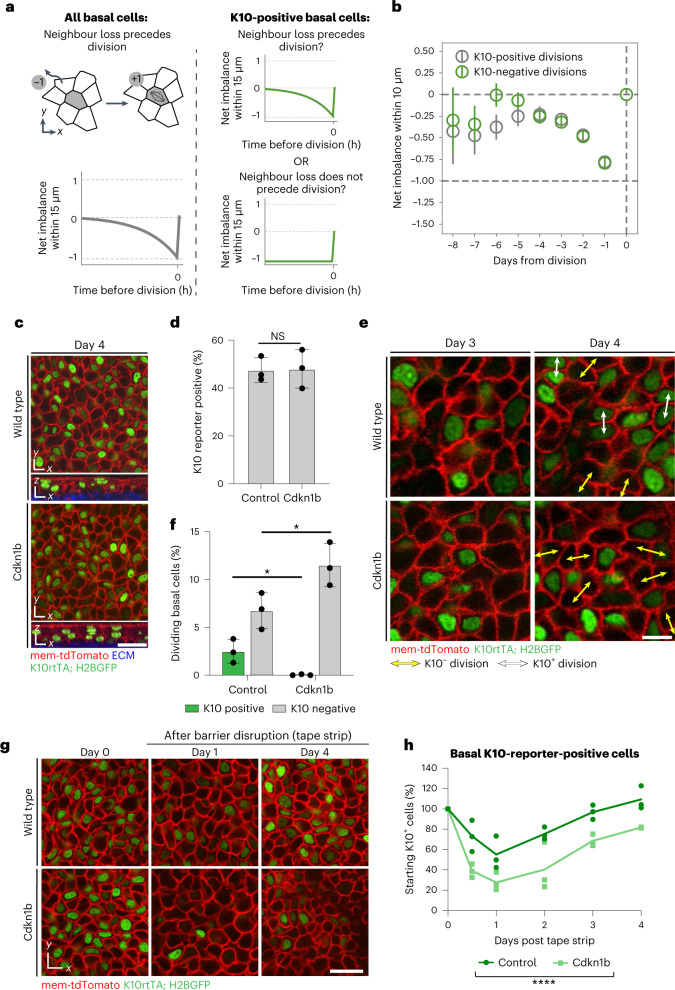
Contributions of differentiating cell proliferation to tissue homeostasis and recovery. **a**, Possible neighbour imbalance scenarios in the days preceding K10-reporter-positive divisions. Cumulative neighbour loss through delamination (−1) and neighbour gain through division (+1) was scored in the days leading up to divisions (Methods). If K10-reporter-positive cells, like the basal population as a whole (left), proliferate in response to neighbour loss, imbalance will drop to −1 before division (right, top green line). If K10-reporter-positive cells are unaffected by neighbour loss, imbalance will not decrease before division (right, bottom line). **b**, Fate imbalance leading up to division events. *N* = 306 reporter-negative and 115 reporter-positive cells from two mice. **c**, Control (K10rtTA; pTRE-H2BGFP) and Cdkn1b (K10rtTA; pTRE-H2BGFP; pTRE-Cdkn1b) epidermis after 4 days of doxycycline administration. K10 reporter is shown in green and membrane shown with mem-tdTomato (red). Scale bar, 25 μm. **d**, Percentage of basal cells expressing the K10 reporter in control and Cdkn1b mice after 4 days of doxycycline administration. Student’s two-tailed *t*-test, *P* > 0.05. NS, not significant. Graph represents average of *n* = 3 imaged mice. **e**, Revisited basal regions from control and Cdkn1b mice between day 3 and day 4 of doxycycline administration, showing K10-reporter-negative divisions (yellow arrows) and K10-reporter-positive division (white arrows). Membrane shown with mem-tdTomato (red). Scale bar, 10 µm. **f**, Percentage of basal cells undergoing K10-reporter-positive and K10-reporter-negative divisions in control and Cdkn1b mice between day 3 and day 4 of doxycycline administration. Student’s two-way *t*-test comparing K10-reporter-positive divisions (green bars, *P* = 0.027) and K10-reporter-negative divisions (grey bars, *P* = 0.046). Graph represents average of *n* = 3 imaged mice. **g**, Revisited basal regions from control and Cdkn1b epidermis before and after barrier disruption via tape stripping. Membrane shown with mem-tdTomato (red). Scale bar, 10 µm. **h**, K10-reporter-positive basal cells within 200 µm × 200 µm basal regions following tape stripping in control and Cdkn1b mice, normalized to day 0 values. ANOVA for linear models, *F*-test *P* = 2 × 10^−6^. For **d**, **f** and **h**, *n* = 2 regions (200 µm × 200 µm) per mouse from at least three mice per genotype, and error bars are mean ± s.d. Source data

**Extended Data Fig. 8:**
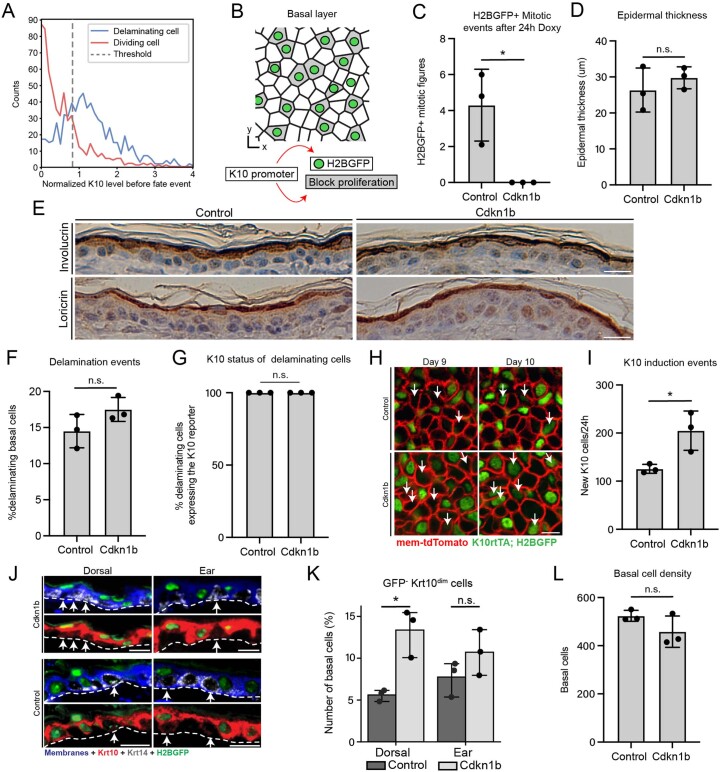
Characterization of K10rtTA; pTRE-Cdkn1b mice. **(A)** Threshold used to define K10 reporter positivity for fate imbalance analysis (Fig. 4A, B). Red line indicates normalized K10 reporter levels in cells one timepoint (24 h) prior to division; blue line indicates K10 reporter levels one timepoint prior to delamination. **(B)** Strategy to block proliferation in differentiating basal cells. K10 promoter-controlled rtTA was used to drive expression of both the G1 cell cycle inhibitor Cdkn1b (pTRE-Cdkn1b) and a fluorescent reporter (pTRE-H2BGFP) in a Doxycycline-inducible manner. All Cdkn1b experiments were performed in ear epidermis. **(C)** K10 reporter positive mitotic figures within 200 µm × 200 µm basal regions after 24 hours of Doxycycline. Student’s two-sided t-test, p < 0.020. **(D)** Average epidermal thickness after 4 days of Doxycycline administration. Student’s two-sided t-test, p > 0.05. **(E)** Involucrin (top row) and Loricrin (bottom row) staining after 10 days of Doxycycline. **(F)** Proportion of basal cells undergoing delamination between Day 3 and Day 4 of Doxycyline. Student’s two-sided t-test, p > 0.05. Scale bars=15 µm. **(G)** Percent tracked cells scored as K10 reporter positive at the timepoint prior to delamination between Day 3 and Day 4 of Doxycyline administration. Student’s two-sided t-test, p > 0.05. **(H)** New K10 induction events, indicated by white arrows, between 9 and 10 days of Doxycycline administration. Scale bar = 10 µm**. (I)** Average number of new K10 induction events within 500 µm x 500 µm basal regions between 9 and 10 days of Doxycycline administration. Student’s two-sided t-test, p < 0.031. **(J)** smFISH staining for *Krt10* and *Krt14* mRNA together with IHC for H2BGFP and a membrane stain in both dorsal and ear skin. Note that due to extremely high levels of *Krt10* mRNA expression in suprabasal and delaminating cells, they look overexposed when increasing channel brightness in order to show *Krt10*-dim cells. Arrows indicate *Krt10*-dim H2BGFP-negative cells. Dashed line marks the basement membrane. Scale bars = 15 µm. **(K)** H2BGFP-negative *Krt10*-dim basal cells in dorsal and ear tissue. Graph represents average from one independent immunostaining experiment using n = 3 mice. Student’s two-sided t-test, p = 0.011 (dorsal), p > 0.05 (ear). **(L)** Average basal density within 200 µm x 200 µm regions after 4 days of Doxycycline administration. Graph represents average of n = 3 imaged mice. Student’s two-sided t-test, p > 0.05. For bar graphs in (**C**), (**D**), (**F**), (**G**), (**I**), (**K**) and (**L**), data is from n = 3 mice and error bars are mean ±S.D. Source data

We next sought to understand whether differentiating cells, just like their undifferentiated neighbours, divide owing to basal layer density changes when nearby cells delaminate^25^ (Fig. 4a). To test this, we quantified the behaviours taking place within 10 μm (a one-cell distance) preceding K10-reporter-positive division events. If these divisions occur in response to a neighbouring delamination, they should be preceded by the net loss of one nearby cell. If K10^+^ divisions occur cell autonomously, or in response to other cues, this imbalance will not be observed (Fig. 4a). Notably, a clear net loss of one neighbour preceded K10^+^ divisions, just like those of K10^−^ cells, indicating that differentiating basal cells indeed divide as a response to delamination in their local neighbourhood (Fig. 4b and Extended Data Fig. 8a).

To directly test the role of K10^+^ divisions during epidermal homeostasis, we used the *Krt10* promoter to induce the cell cycle inhibitor *Cdkn1b* (or *p27*) (ref. ^26^) in differentiating cells and monitored these cells with the K10 reporter (*K10rtTA*; *pTRE-Cdkn1b; pTRE-H2BGFP*) (Extended Data Fig. 8b). CDKN1B induction caused a rapid and penetrant block of proliferation in K10^+^ cells (Extended Data Fig. 8c) but did not alter epidermal thickness or expression of terminal markers (Extended Data Fig. 8d,e). The number of delaminating cells was comparable in tissue with and without CDKN1B induction, and all cells acquired K10 reporter expression before delamination regardless of genotype (Extended Data Fig. 8f,g), demonstrating that cell division is not required for the later maturation nor delamination of differentiating cells. Most interestingly, expression of CDKN1B did not affect the number of K10-reporter-positive basal cells (Fig. 4c,d), indicating that the size of the differentiating cell pool is maintained even when these cells are entirely unable to divide. Notably, the number of new K10-reporter-positive cells that emerged over a 24 h period was significantly higher in Cdkn1b mice (Extended Data Fig. 8h,i), and the number of basal cells expressing *Krt10*^*dim*^ mRNA within the K10-reporter-negative population was increased (Extended Data Fig. 8j,k), suggesting a more rapid onset of differentiation-associated transcriptional changes in these mice. Thus, in the absence of amplifying K10 divisions, an increased number of basal cells initiate differentiation to maintain the size of the differentiating cell pool.

To next understand how the density of the basal layer was maintained even after 4 days of CDKN1B induction (Extended Data Fig. 8l), we directly tracked the number of cell divisions taking place in Cdkn1b versus control tissue over a 24 h period. We found that K10^−^ basal cells in the Cdkn1b epidermis increased their proliferation rate to equal the number of divisions performed by both K10^+^ and K10^−^ cells in control tissue (Fig. 4e,f). These results indicate that the homeostatic need for proliferation is normally satisfied by the contribution of both differentiating and undifferentiated cells. Thus, proliferation of differentiating cells in the adult epidermis occurs as a consequence of the need to replace delaminating basal neighbours, and not as part of an obligate transit-amplifying programme that fuels proper numbers of differentiating cells.

**Extended Data Fig. 9:**
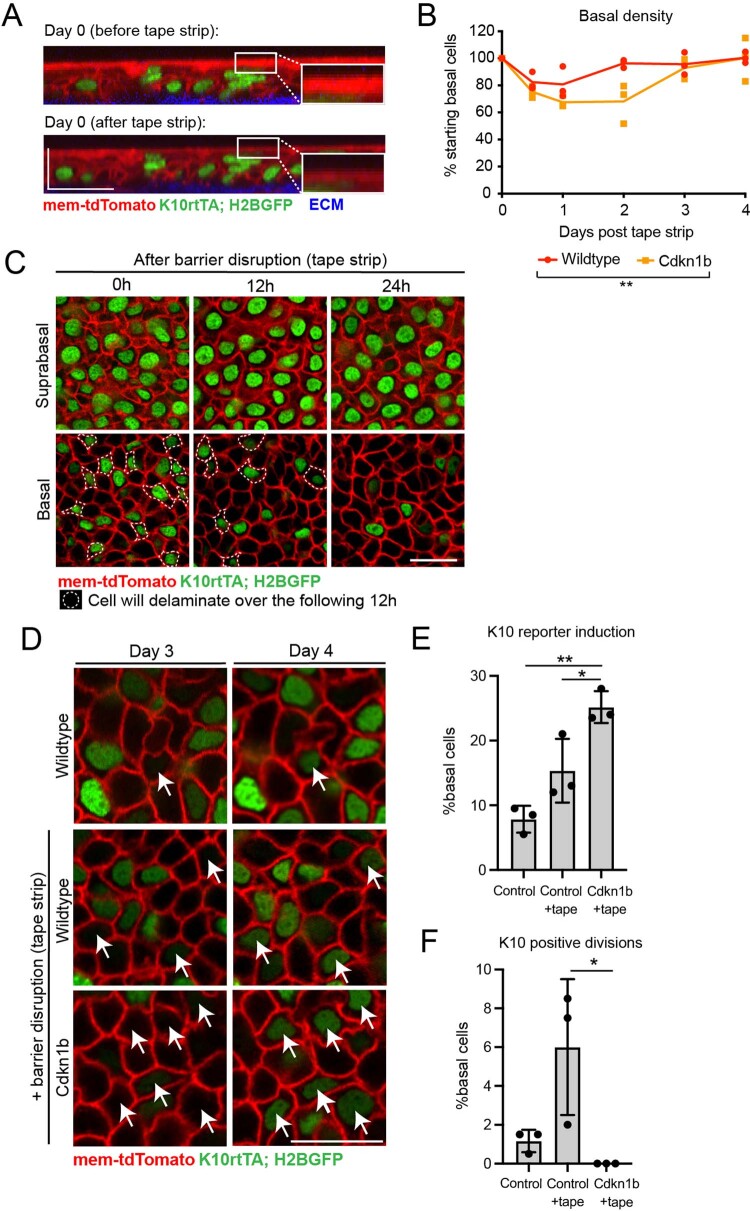
Characterization of control and Cdkn1b tissue after epidermal barrier disruption. **(A)** Transverse images of the same epidermal region from the ear of a *K10rtTA; pTRE- H2BGFP; mTmG* mouse before and directly after tape stripping to disrupt the epidermal barrier. Insets show a close-up view of the epidermal surface before and after removal of the outermost cornified cells, accompanied by visible thinning of *mTmG* signal. Images are representative of n = 3 mice. **(B)** Normalized basal cell density within 200 µm × 200 µm regions in the days following tape stripping in control and Cdkn1b mice, normalized to Day 0 values. ANOVA for linear models, F-test p = 0.006742 **(C)** Revisited basal and suprabasal regions at 0 h, 12 h and 24 h after tape stripping. Dotted lines indicate cells that will delaminate out of the basal layer in the subsequent 12 h. Scale bar = 10 µm. Images are representative of n = 3 mice. **(D)** Revisited basal regions from Control mice during homeostasis (top row) and Control and Cdkn1b after tape stripping (bottom rows). White arrows indicate new K10 induction events. Scale bar = 10 µm. **(E)** Proportion of basal cells inducing K10 reporter expression in Control ear skin during homeostasis and Control and Cdkn1b ear skin after tape stripping. Student’s two-sided t-test, p < 0.05 (Control + tape vs Cdkn1b vs tape); p < 0.01 (Control vs Cdkn1b vs tape). Graph represents average of n = 3 imaged mice per condition. **(F)** Proportion of basal cells undergoing K10 positive divisions Control ear skin during homeostasis and Control and Cdkn1b ear skin after tape stripping. Student’s two-sided t-test, p < 0.05 (Control + tape vs Cdkn1b vs tape), graph represents average of n = 3 imaged mice per condition. For bar graphs in (**E**) and (**F**), error bars represent S.D. Source data

Finally, we evaluated the behaviours of differentiating basal cells in response to disruption of the epidermal barrier using tape stripping (Extended Data Fig. 9a). This perturbation caused a reduction of basal cell density (Extended Data Fig. 9b) due to a 40% depletion of K10^+^ basal cells through a rapid wave of delaminations within 24 h (Fig. 4g,h and Extended Data Fig. 9c). Notably, the differentiating basal pool in wild-type epidermis was quickly replenished to homeostatic levels within 3 days (Fig. 4g,h) through a combination of increased K10^+^ cell divisions and increased de novo K10 expression (Extended Data Fig. 9d–f). We observed a more severe initial reduction of the basal cell density and basal K10^+^ population when we performed the same perturbation in Cdkn1b epidermis (Fig. 4g,h and Extended Data Fig. 9b), indicating that K10^+^ cell divisions help to initially buffer the basal layer from excessive K10 cell loss. Consequently, Cdkn1b tissue was delayed in its ability to recover this population compared with wild-type epidermis (Fig. 4h and Extended Data Fig. 9b), and more undifferentiated basal cells initiated K10 expression in Cdkn1b tissue than in wild-type controls (Extended Data Fig. 9d,e). Thus, proliferation of K10^+^ basal cells helps replenish the differentiating cell pool during recovery after barrier disruption.

In summary, this study demonstrates the continuous, progressive and multi-day nature of the transcriptional changes associated with basal cell differentiation, reveals the cell-to-cell variability in the timing and behaviours associated with this process, and indicates that the onset of K10 expression both precedes downregulation of stem cell genes and marks cells committed to leaving the basal layer. We also show that the initiation of differentiation is both temporally and functionally uncoupled from cell cycle exit, with a subset of K10^+^ cells undergoing proliferation in response to local density changes. When this proliferative capacity is blocked, differentiating cells remain capable of maturing and stratifying normally, and the nearby stem cells compensate through increased rates of proliferation and differentiation. Even upon acute barrier disruption, the differentiating population recovers with only a mild delay when K10^+^ divisions are blocked.

Despite representing a robust marker of early basal cell differentiation, K10-deficient epidermis stratifies normally and forms a well-developed barrier^27,28^. Previous work has indicated highly complex transcriptional regulation of K10 expression via factors such as the TFAP2 and CEBP TF families and the Notch pathway^29–34^. Using SCENIC^35^ to analyse potential regulons, we identified several TFs that could play an important role in initiating differentiation and/or inhibiting stemness-related genes (Extended Data Fig. 6 and Methods), including *Cebpb*, *Bhlhe40*, *Hes1*, *Id1*, *Ovol1* and *Trp63*^19,31,36–38^, and the interesting additional candidate *Csnk2b*, which plays a role in astrocyte differentiation^39^. Future studies that combine in vivo imaging of K10 dynamics with more precise spatial and temporal manipulation will help to unravel the upstream mechanisms driving epidermal stem cell differentiation.

Our results demonstrate that all delaminating cells transition through a K10-positive state in which they retain a limited capacity to divide, but proliferation is not necessary for their further maturation or stratification. This argues against the presence of a long-lived and functionally distinct committed epidermal progenitor population. At the same time, we reveal direct evidence of a molecular heterogeneity among the proliferating basal population. This helps to clarify the distinct results obtained by different lineage tracing approaches^1–4^, and explains the strong temporal correlation in lifetimes between sister cells that both go on to delaminate^10^. Taken together, our results reveal that a single continuous differentiation process shaped by feedback from the local environment fuels epidermal turnover.

## Methods

### Mice and experimental treatment

*mTmG*^40^, *K14-CreER*^41^, *tetO-Cdkn1b*^26^, *Ivl-CreERT2* (ref. ^42^) and *R26-tdTomato*^43^ mice were obtained from the Jackson Laboratory. *K10-rtTA*^15^ mice were obtained from T. Lechler (Duke University), *pTRE-H2BGFP*^44^ mice were obtained from E. Fuchs (Rockefeller University), *Lifeact-GFP*^45^ mice were obtained from R. Weigert (National Institute of Dental and Craniofacial Research, National Institutes of Health (NIH)), *GFP-NMMIIB*^46^ mice were obtained from R. Adelstein (National Heart, Lung, and Blood Institute, NIH) and *R26p-Fucci2* (ref. ^47^) mice were obtained from S. Aizawa (RIKEN). *K14-H2BmCherry*^48^ mice were generated in the laboratory and described previously. To visualize clonally labelled basal cells as they delaminate, *K14CreER; mTmG* mice were given a single dose of tamoxifen (20 μg/g in corn oil) 3 days before imaging. *IvlCreERT2; R26-Tomato* mice were treated with tamoxifen once at 8 weeks (0.2 mg g^−1^ in corn oil intra-peritoneally; Sigma Aldrich, catalogue number T5648), 2 days before cell isolation. To visualize K10 expression, *K10-rtTA*; *pTRE-H2BGFP*; *mTmG* mice were given doxycycline (2 mg ml^−1^) in drinking water with 2% sucrose continuously, starting 3 days before imaging. To block proliferation in the K10-positive population, littermates were genotyped to identify mutant (*K10-rtTA*; *pTRE-H2BGFP*; *mTmG; tetO-Cdkn1b)* and control (*K10-rtTA*; *pTRE-H2BGFP*; *mTmG)* mice. Animals were given doxycycline (2 mg ml^−1^) in drinking water with 2% sucrose continuously for the times specified. To disrupt the epidermal barrier via tape stripping, standard coloured lab tape (Fisher Scientific) was applied to the surface of the ear skin and removed in ten sequential repetitions. All mice used in this study were between 6 and 10 weeks old and were maintained either on a CD1 background (intra-vital imaging) or C57BL/6J background (single-cell RNA sequencing, scRNA-seq). Mice from experimental and control groups were randomly selected from either sex for live imaging experiments. Data collection and analysis were not performed blind to the conditions of the experiments. All procedures involving animal subjects were performed under the approval of the Institutional Animal Care and Use Committee of the Yale School of Medicine or the Linköping Animal Ethics Committee in accordance with Swedish legislation.

### In vivo imaging

All imaging was performed in distal regions of the ear skin during prolonged telogen, with hair removed using depilatory cream (Nair) ≥3 days before the start of each experiment. Mice were anaesthetized with vapourized isofluorane delivered by a nose cone. Image stacks were acquired with a LaVision TriM Scope II (LaVision Biotec) laser scanning microscope equipped with both a Chameleon Vision II and Discovery two-photon lasers (Coherent). For collection of serial optical sections, the laser beam was focused through a 40× water immersion lens (Nikon; numerical aperture 1.15) and scanned with a field of view of 0.3 mm × 0.3 mm at 600 Hz. *z*-Stacks were acquired with 0.5–1 µm steps to image a total depth of ~40 µm of tissue, covering the entire thickness of the epidermis. Visualization of ECM was achieved via second harmonic signal using the blue channel at 940 nm imaging wavelength. To follow the same epidermal cells over multiple days, inherent landmarks of the skin together with a micro-tattoo were used to navigate back to the same epidermal regions every 12 or 24 h. For time-lapse imaging, serial optical sections were obtained in a range of 5–8 min intervals for a total duration of 1–3 h.

### Image analysis

For quantifications in Figs. 1, 3, and 4c–h and Extended Data Figs. 1–5 and 7–9, raw image stacks were imported into Fiji^49,50^ for further analysis. Basal footprint and suprabasal spreading were quantified by manually outlining cell boundaries directly above the ECM signal and at the widest section in the top half of each cell, respectively. K10 reporter levels were quantified by measuring H2BGFP signal intensity at the widest plane of each cell nucleus. All values shown in this paper represent absolute fluorescence (with the exception of Fig. 4b; see below), and the same cut-off for positivity was applied to all cells within each experiment (1,500 for Figs. 1 and 3; 2,000 for Fig. 4c–h; for details of threshold for Fig. 4b, see below). K10 protein levels in whole-mount images were quantified by measuring average cytoplasmic signal intensity at the midpoint of each cell. Cell behaviours were tracked by visually comparing epidermal regions at subsequent timepoints, and cells were scored as suprabasal at the first timepoint when they made no observable contact with the underlying ECM signal. Prism software (GraphPad) was used to graph data and perform statistical analysis.

To track dynamics in the basal layer for Fig. 4b, we adapted the procedure from ref. ^25^. To height correct the 3D images, we first Gaussian blurred the signal from the ECM (mouse 1) or the epidermal cell nuclei (K14H2BmCherry) (mouse 2) spatially in the *xy* plane (width 6 μm) to create a 3D mask representing the region covering the whole epidermis. We then defined the height of the interface between the epidermis and the dermis from the 3D mask and subtracted this height from the original 3D data to level the basal layer position. From the height-corrected 3D images, we took three consecutive z-positions containing the nuclei of all the basal layer cells and averaged the intensity over the three slices to obtain 2D images in each channel. We calculated the local minima or maxima of the epithelial cell membranes (mem-tdTomato) or epithelial cell nuclei (K14H2BmCherry) to represent the cell positions in mouse 1 or mouse 2, respectively, and automatically corrected the shifts between time frames by minimizing the square distance between the nearest cell positions across the frames. The intensity of K10 reporter in the nucleus was calculated by taking the mean of the H2BGFP signal within a circle of 3 μm radius around the cell position (mouse 1) or by taking the sum within the segmented cell regions (mouse 2). After dividing the K10 signal levels by the mean within dividing cells for each region, we manually set a threshold to define cell divisions with high K10, as shown in Supplementary Fig. 8A (13 out of 49 cell divisions in mouse 1, 102 out of 374 cell divisions in mouse 2).

To assess neighbour fate imbalance before cell division, we took the sum over the net imbalance of the fates in the frames leading up to each division event. The net imbalance was calculated by taking the timepoint right after the division as zero, and going back in time while adding one (differentiation) or subtracting one (division) whenever there was a fate event within in the 10 μm neighbourhood of the division event of interest. We then averaged this net imbalance track over all the division events in mice 1 and 2. The error bar represents the fluctuation of net imbalance over each division event.

Single-molecule FISH image quantification was performed with the Cell Counter plugin in Fiji, and results were analysed with custom Python scripts. All quantifications were performed on *n* = 3 biological replicates.

### Immunostaining

To isolate epidermis for whole-mount staining, ear and dorsal skin were incubated in 5 mg ml^−1^ dispase II solution (Sigma, 4942078001) at 37 °C for 10 min or 4 °C overnight, respectively, and the epidermis was separated from dermis using forceps. Epidermal tissue was fixed in 4% paraformaldehyde in PBS for 45 min at room temperature, washed in PBS, permeabilized and blocked for >1 h (0.2% Triton-X, 5% normal donkey serum and 1% BSA in PBS) and then incubated in primary antibodies overnight at 4 °C and secondary antibodies for 3 h at room temperature. For histological analysis of terminal differentiation markers, 10% formalin-fixed, paraffin-embedded (FFPE) skin was cut in 5 μm sections. Primary antibodies used were as follows: rabbit anti-K10 (1:1,000; Biolegend Poly19054), guinea pig anti-K10 (1:400; Progen GP-K10), rabbit anti-pH3 (1:1,000; Millipore 06-570), chicken anti-GFP (1:1,000; Invitrogen A10262), rabbit anti-involucrin (1:750; Biolegend Poly19244) and rabbit anti-loricrin (1:1,000; Biolegend Poly19051). All secondary antibodies used for immunofluorescence were raised in a donkey host and were conjugated to AlexaFluor 488, 568 or 647 (1:400; Thermo Fisher A78950, A21206, A10042, A31573, A21202, A10037 or A31507). When used, AlexaFluor 647 Phalloidin (Thermo Fisher) was incubated at the same time as secondary antibodies. 5-Ethynyl-2′-deoxyuridine (EdU) was administered via intra-peritoneal injection (50 μg g^−1^ in PBS) 2 h before collecting tissue, and EdU labelling was performing using the Click-iT AlexaFluor 568 kit (Thermo Fisher) according to the manufacturer’s instructions. Fixed whole-mount tissue was mounted on a slide with Vectashield anti-fade mounting medium (Vector Laboratories) with a #1.5 coverslip. For brightfield immunohistochemistry, biotinylated species-specific secondary antibodies followed by detection using the ABC kit (Vector Labs) and DAB kin (Vector Labs) were used according to the manufacturer’s instructions.

### FISH

smRNA-FISH was performed using the RNAscope Multiplex Fluorescent Detection Kit v2 (323100, Advanced Cell Diagnostics) according to the manufacturer’s instructions using tyramide signal amplification (TSA) with Cy3, Cy5 and/or fluorescein (NEL760001KT, Perkin Elmer) on FFPE sections of dorsal skin. FFPE sections were hybridized with combinations of the following mRNA probes (all from Advanced Cell Diagnostics): Krt10 (457901 and 457901-C2), Krt14 (422521-C3), Krt5 (547901), Mt4 (447121-C3) and Krtdap (500671), together with cell membrane counterstaining using WGA (1:50, 29028-1 and 29059-1, Biotium). RFP (1:100, 600-401-379, Rockland) and GFP (1:200, ab13970, Abcam or 1:100 Cell Signaling 2965) immunohistochemistry was performed together with smRNA-FISH stainings according to manufacturer’s instructions with secondary antibodies that were raised in donkey or goat host and conjugated to AlexaFluor 405, 488, 546 or 647 (1:500; Thermo Fisher A10040, A11008, A21245 or A31556). Stainings were performed on skin samples isolated from the same mice that were used for ITGA6-sorted cell sequencing, wild-type 8-week-old mice, K10rtTA; pTRE-H2BGFP mice, or Cdkn1b mice and their respective controls. Images were acquired on a Nikon A1R spinning disk confocal as tiled images (10–15% overlap) and stitched by NIS Elements. Subsequently, all images were processed in the same way (maximum intensity projection, brightness adjustment and pseudocolouring) using Fiji^36,37^. In the case of Supplementary Fig. 8j, CDKN1B ear sample *Krt10* staining bled through into the *Krt14* channel, which was corrected for by subtracting *Krt10* staining intensities from *Krt14* intensities.

### scRNA-seq and library preparation

For the scRNA-seq, epidermal cells were isolated from the back skin of 8-week-old *Ivl*-traced mice as described previously^19^. Cells were stained for 1 h with CD49f (Itga6)-AlexaFluor 647 (1:50; BD Biosciences; catalogue number 551129), Sca1(Ly6a)-PeCy7 (1:50; BD Biosciences; catalogue number 558162) and Cd34-FITC (1:50; BD Pharmingen catalogue number 553733) and Sytox blue (1:1,000; Life Technologies catalogue number S34857) was added just before (2 min before) FACS sorting on FACSAria III machine with BD FACSDiva 8.0.1 software (BD Biosciences). Tomato-traced and non-traced live cells gated for ITGA6^+^/SCA1^+^/CD34^−^ were collected in 400 μl Defined Keratinocyte serum-free medium (Thermo Fisher, catalogue number 10744019) with 1% DNaseI (Stem Cell Technologies, catalogue number 07900), loaded into Fluidigm C1 chips (Fluidigm) for library preparation and sequenced as described previously^19^.

### Data analysis

Pre-processing of the sequencing results into count matrices was performed as in ref. ^19^, and all subsequent data analysis was performed using Scanpy^51^. From the previously published datasets^19,20^ only cells that were categorized as ‘interfollicular epidermis cells’ (IFE) were included. Each dataset (ITGA6 sorted, Joost 2016 and Joost 2020 main dataset) was separately normalized using size factors and logarithmitized (ln(*X* + 1)), before filtering out non-expressed genes. Further, each dataset was regressed for the effects of total counts per cell, percentage of External RNA Controls Consortium (ERCC) spike-in counts for the ITGA6-sorted and Joost 2016 datasets (Fluidigm C1 based), and cell cycle (as scored by ‘score_genes_cell_cycle’ method). To combine all data, we first merged the C1-based datasets using the shared genes from the top 4,000 highly variable genes from each individual dataset. For integrating the Joost 2020 dataset, we used genes present in the C1-merged data. To compensate for the lower per-cell read counts in the Joost 2020 dataset (10x Chromium based), Scanpy implementation of MAGIC imputation^52^ was used with the following parameters specified: *k*-nearest neighbour (kNN) = 10; *t* = 2. This merged and imputed dataset revealed a small outlying group of cells (*n* = 9 cells) and a population of infundibulum cells (*n* = 147 cells), which were removed from subsequent analysis (Jupyter notebook). Cell cluster classification was performed on the imputed data using the previously defined differentiation trajectory from Joost 2016 as a reference. A kNN classifier (Scikit-learn^53^) was used to assign the cluster identity of cells from the ITGA6-sorted and Joost 2020 datasets according to the reference dataset with the following parameters specified: kNN = 20; weight = ‘distance’. For gene expression analyses, raw count matrices were log-normalized and downsampled to 2,000 counts per cell, compensating for the higher read counts in Fluidigm C1-based datasets, and merged. Pseudotime analysis was performed using diffusion pseudotime implementation in Scanpy^51,54^ and by ordering cells on the basis of their position along the pseudotime. The cut-off for basal and suprabasal populations was defined by the 95th percentile of ITGA6-sorted cells on the ordered differentiation pseudotime. The cut-off for cells to be classified as *Krt10* positive was defined by the mean expression of *Krt10* in all cells (1.84 log-normalized counts). For further analysis of gene expression over pseudotime, cells were grouped into six and four equally sized bins for basal and suprabasal populations, respectively. Fitted gene expression trends along the pseudotime were compared with the average expression levels in bin 1 (considered to be the most basal) and plotted as the change (log normalized) compared with this baseline expression. Spliced and unspliced mRNA analysis was performed on the annotated dataset from Joost 2020 (mapped with Velocyto package^55^). To avoid any bias due to sequencing methods used and by merging the datasets, cell cycle analysis was performed on the largest dataset (before regression), covering males and females of different ages^20^. Cell cycle phase was assigned with ‘score_genes_cell_cycle’ using a cut-off of 0.05 for positive classification. For mapping the Aragona et al. 2020 dataset onto our differentiation timeline, only those cells defined in Aragona et al. as ‘basal IFE’, ‘cycling IFE’ and ‘suprabasal IFE’ were included (log-normalized and subset to include only genes that are expressed in all datasets). Finally, each dataset was independently scaled to unit variance and zero mean before mapping the Aragona et al. dataset to our combined uniform manifold approximation and projection (UMAP) using ingest implementation in Scanpy.

### SCENIC analysis

SCENIC analysis was performed using the pySCENIC (v0.11.2) Python package together with Scanpy (v1.7.2) and loompy (v3.0.6) for data processing and visualization. Overall, the analysis was performed as described in the full SCENIC analysis example and the extended analysis examples with default parameters. Network inference was performed with GRNBoost2 algorithm using the downsampled dataset from our analysis and the murine TF list (mm_mgi_tfs.txt) supplied by SCENIC. Regulon and motif prediction was done within 10 kb of the transcription start site, using the following databases (from cisTarget): mm10__refseq-r80__10kb_up_and_down_tss.mc9nr.feather and motifs-v9-nr.mgi-m0.001-o0.0.tbl. Unfortunately, we did not find enriched motifs with a clear link to differentiation (for example, targeting *Krt10*) during the downstream analysis.

To identify differentiation associated TFs and their potential target genes, we performed the following analysis. (1) Find genes that are correlated with *Krt10* expression (Pearson *r* > 0.3) during the earliest differentiation steps (bins 2, 3 and 4). (2) Identify TFs that target any of these differentiation-associated genes (based on the adjacency list from GRNBoost2 analysis), and include genes that are targeted by the same TF. (3) Give each cell a score based on the expression levels of the target genes for each of the TF (using scanpy.tl.score_genes() function). Note that this step in SCENIC analysis includes genes that are both up- and downregulated during the differentiation process (that is, TF expression levels can be used to predict the expression of the gene), so the resulting target gene list also included several basal-layer-associated genes (that is, *Krt14*, *Krt5*, *Col17a1*, *Mt2* and so on). To show only the relationships between TF and differentiation, we first identified differentially expressed genes (using scanpy.tl.rank_genes_groups()) in bin 1 (adjusted *P* value < 0.05, log fold change >0.5) and excluded them from gene scoring.

### Statistics and reproducibility

Statistical parameters including the exact value of *n* for each experiment and statistical significance are reported in figure legends. Significance was determined using an unpaired Student’s *t*-test, pairwise Tukey’s honest significant difference (HSD) when multiple comparisons were made or analysis of variance (ANOVA) for the time-series analysis (see below). Asterisks denote statistical significance (*P < 0.05, **P < 0.01, ***P < 0.001 and ****P < 0.0001). Statistical calculations were performed using the Prism software package (GraphPad), Scanpy for differential gene expression analysis or statsmodels (v0.12.2) library in Python for time-series analysis. No statistical methods were used to pre-determine sample sizes, but our sample sizes are similar to those reported in previous publications^10,19,20,25^. All figures represent data from at least three independent experiments from at least *n* = 3 mice, except Figs. 1b,c and 3h and Extended Figs. 1e, 7g and 8k, which represent data from one independent experiment with *n* = 3 mice.

Comparison of the effects of Cdkn1b mouse model on the number of K10^+^ basal cells (Fig. 4h) and basal cell density (Supplementary Fig. 9b) after tape stripping was performed as follows. First, cubic linear models were constructed (statsmodels.regression.linear_model.OLS) to explain the observed proportion of cells depending either only on the day after tape stripping (H_0_) or on a combination of the day and genotype (H_1_):

$${{{\mathrm{H}}}}_0:{Y}\sim {X} + {X}^2 + {X}^3$$

$${{{\mathrm{H}}}}_1:{Y}\sim \left( {{X} + {X}^2 + {X}^3} \right) \times {G}$$

where *Y* is proportion of cells, *X* is day after tape stripping and *G* is genotype.

Subsequently, ANOVA for linear models (statsmodels.stats.anova.anova_lm) was used to compare if the model with the addition of genotype (H_1_) is better at explaining the observed data than the model using only days (H_0_), using the default *F*-test.

### Reporting summary

Further information on research design is available in the Nature Research Reporting Summary linked to this article.

## Online content

Any methods, additional references, Nature Research reporting summaries, source data, extended data, supplementary information, acknowledgements, peer review information; details of author contributions and competing interests; and statements of data and code availability are available at 10.1038/s41556-022-01021-8.

## Supplementary information

Reporting Summary

Supplementary Video Time-lapse recording over 88 min of a representative K10-reporter-positive basal cell dividing to produce two basal daughter cells. Top shows *xz* view, and bottom shows *xz* view of the same cell. K10 reporter signal (*K10rtTA; pTRE-H2BGFP*) shown in green, cell membrane (mem-tdTomato) shown in red and dermal ECM shown in blue. Scale bar, 5 μm.

## Data Availability

Sequencing data that support the findings of this study have been deposited in the Gene Expression Omnibus (GEO) under accession code GSE152044. Previously published scRNA-seq data that were re-analysed here are available under accession codes GSE129218, GSE67602 and GSE146637. Annotated and analysed sequencing data have been deposited in Zenodo: 10.5281/zenodo.6998285. Source data are provided with this paper. All other data supporting the findings of this study are available from the corresponding authors on reasonable request.

## Source data

Source Data Fig. 1 Statistical source data.
Source Data Fig. 3 Statistical source data.
Source Data Fig. 4 Statistical source data.
Source Data Extended Data Fig. 1 Statistical source data.
Source Data Extended Data Fig. 2 Statistical source data.
Source Data Extended Data Fig. 3 Statistical source data.
Source Data Extended Data Fig. 4 Statistical source data.
Source Data Extended Data Fig. 7 Statistical source data
Source Data Extended Data Fig. 8 Statistical source data.
Source Data Extended Data Fig. 9 Statistical source data.

## Code availability

The complete computational analysis workflow for the scRNAseq is available in the form of Jupyter notebooks at https://github.com/kasperlab. The Python scripts for the image analysis will be available on request.
