# Supplementary material for: Gradual differentiation uncoupled from cell cycle exit generates heterogeneity in the epidermal stem cell layer
Source: Nat Cell Biol. 2022 Nov 10;24(12):1692–700. doi: 10.1038/s41556-022-01021-8 (PMC9729105; doi:10.1038/s41556-022-01021-8)
Supplement: Supplementary file 1 — Reporting Summary [file 41556_2022_1021_MOESM1_ESM.pdf]

nature portfolio

Corresponding author(s): Maria Kasper and ValentinaGreco

Last updated by author(s): Aug 19, 2022

Reporting Summary

Nature Portfolio wishes to improve the reproducibility of the work that we publish. This form provides structure for consistency and transparency in reporting. For further information on Nature Portfolio policies, see our Editorial Policies and the Editorial Policy Checklist.

Statistics

For all statistical analyses, confirm that the following items are present in the figure legend, table legend, main text, or Methods section.

n/a	Confirmed	
☐	☒	The exact sample size (n) for each experimental group/condition, given as a discrete number and unit of measurement
☐	☒	A statement on whether measurements were taken from distinct samples or whether the same sample was measured repeatedly
☐	☒	The statistical test(s) used AND whether they are one- or two-sided<br>*Only common tests should be described solely by name; describe more complex techniques in the Methods section.*
☒	☐	A description of all covariates tested
☐	☒	A description of any assumptions or corrections, such as tests of normality and adjustment for multiple comparisons
☐	☒	A full description of the statistical parameters including central tendency (e.g. means) or other basic estimates (e.g. regression coefficient) AND variation (e.g. standard deviation) or associated estimates of uncertainty (e.g. confidence intervals)
☐	☒	For null hypothesis testing, the test statistic (e.g. F, t, r) with confidence intervals, effect sizes, degrees of freedom and P value noted<br>*Give P values as exact values whenever suitable.*
☒	☐	For Bayesian analysis, information on the choice of priors and Markov chain Monte Carlo settings
☒	☐	For hierarchical and complex designs, identification of the appropriate level for tests and full reporting of outcomes
☐	☒	Estimates of effect sizes (e.g. Cohen's d, Pearson's r), indicating how they were calculated

Our web collection on statistics for biologists contains articles on many of the points above.

Software and code

Policy information about availability of computer code

Data collection	Inspector Pro (LaVision Biotec, v.7.0.129.0), NIS Elements (Nikon), Illumina HiSeq 2000 (Illumina)
Data analysis	Fiji (NIH, v.2.0.3), Prism (GraphPad, v9.3.1), Scanpy (1.6 - 1.8)

For manuscripts utilizing custom algorithms or software that are central to the research but not yet described in published literature, software must be made available to editors and reviewers. We strongly encourage code deposition in a community repository (e.g. GitHub). See the Nature Portfolio guidelines for submitting code & software for further information.

Data

Policy information about availability of data

All manuscripts must include a data availability statement. This statement should provide the following information, where applicable:

- Accession codes, unique identifiers, or web links for publicly available datasets
- A description of any restrictions on data availability
- For clinical datasets or third party data, please ensure that the statement adheres to our policy

Sequencing data that support the findings of this study have been deposited in the Gene Expression Omnibus (GEO) under accession code GSE152044. Previously published scRNA-sequencing data that were re-analysed here are available under accession codes GSE129218, GSE67602 and GSE146637. Annotated and analyzed sequencing data have been deposited in zenodo: HYPERLINK "https://doi.org/10.5281/zenodo.6998285" 10.5281/zenodo.6998285.

Source data are provided with this study. All other data supporting the findings of this study are available from the corresponding authors on reasonable request.

Human research participants

Policy information about studies involving human research participants and Sex and Gender in Research.

Reporting on sex and gender	Not applicable.
Population characteristics	Not applicable.
Recruitment	Not applicable.
Ethics oversight	Not applicable.

Note that full information on the approval of the study protocol must also be provided in the manuscript.

Field-specific reporting

Please select the one below that is the best fit for your research. If you are not sure, read the appropriate sections before making your selection.

☒ Life sciences ☐ Behavioural & social sciences ☐ Ecological, evolutionary & environmental sciences

For a reference copy of the document with all sections, see nature.com/documents/nr-reporting-summary-flat.pdf

Life sciences study design

All studies must disclose on these points even when the disclosure is negative.

Sample size	No statistical methods were used to pre-determine sample sizes but our sample sizes are similar to those reported in previous publications (Rompolas, Mesa et al. 2016, Mesa, Kawaguchi, Cockburn et a. 2018; Joost et al. 2016, Joost, Annusver et al. 2020).
Data exclusions	No data were excluded from the analysis.
Replication	Experiments were replicated at least three times unless otherwise specified. Results from replicates show consistency.
Randomization	Mice from the same litter were designated as experimental (Cdkn1b positive) or control (Cdkn1b negative) based on genotype.
Blinding	Investigators were not blinded during data collection and analysis. Blinding was not possible since the main researchers were responsible for both data acquisition and analysis for many experiments.

Reporting for specific materials, systems and methods

We require information from authors about some types of materials, experimental systems and methods used in many studies. Here, indicate whether each material, system or method listed is relevant to your study. If you are not sure if a list item applies to your research, read the appropriate section before selecting a response.

Materials & experimental systems

n/a	Involved in the study	
☐	☒	Antibodies
☒	☐	Eukaryotic cell lines
☒	☐	Palaeontology and archaeology
☐	☒	Animals and other organisms
☒	☐	Clinical data
☒	☐	Dual use research of concern

Methods

n/a	Involved in the study	
☒	☐	ChIP-seq
☐	☒	Flow cytometry
☒	☐	MRI-based neuroimaging

Antibodies

Antibodies used	Primary antibodies used were as follows: rabbit anti-K10 (1:1000; Biolegend Poly19054), guinea pig anti-K10 (1:400; Progen GP-K10), rabbit anti-pH3 (1:1000; Millipore 06-570), chicken anti-GFP (1:1000; Invitrogen A10262), rabbit anti-involucrin (1:750, Biolegend Poly19244), rabbit anti-loricrin (1:1000, Biolegend19051), rabbit anti-RFP (1:100, Rockland 600-401-379), rabbit anti-GFP (1:100, Cell Signalling 2965) and chicken anti-GFP (1:200, Abcam ab13970). All secondary antibodies used were raised in a donkey or goat host

	and were conjugated to AlexaFluor 405, 488, 546, 568 or 647 (1:400 or 1:500; Thermofisher A31556, A78950, A21206, A10042, A31573, A21202, A21245, A10037, A10040, or A31507).
Validation	Antibody validation information can be found on manufacturers' websites. Rabbit anti-K10 (https://www.biolegend.com/en-us/products/purified-anti-keratin-10-antibody-13377), guinea pig anti-K10 (https://www.progen.com/anti-keratin-k10-guinea-pigpolyclonal-serum.html), rabbit anti-pH3 (https://www.emdmillipore.com/US/en/product/Anti-phospho-Histone-H3-Ser10-Antibody-Mitosis-Marker,MM_NF-06-570), chicken anti-GFP (https://www.thermofisher.com/antibody/product/GFP-Antibody-Polyclonal/A10262), rabbit anti-involucrin (https://www.biolegend.com/fr-fr/products/anti-involucrin-antibody-11077?pdf=true&displayInline=true&leftRightMargin=15&topBottomMargin=15&filename=Anti-Involucrin%20Antibody.pdf), rabbit antiloricrin (https://www.biolegend.com/nl-nl/products/purified-anti-loricrin-antibody-13325?pdf=true&displayInline=true&leftRightMargin=15&topBottomMargin=15&filename=Purified%20anti-Loricrin%20Antibody.pdf), rabbit anti-RFP (https://www.rockland.com/categories/primary-antibodies/rfp-antibody-pre-adsorbed-600-401-379/), rabbit anti-GFP (https://www.cellsignal.com/products/primary-antibodies/gfp-d5-1-rabbit-mab/2956) and chicken anti-GFP (https://www.abcam.com/gfp-antibody-ab13970.html).

Animals and other research organisms

Policy information about studies involving animals; ARRIVE guidelines recommended for reporting animal research, and Sex and Gender in Research

Laboratory animals	mTmG (Muzumdar et al, 2007), K14-CreER (Vasioukhin et al, 1999), tetO-Cdkn1b (Pruitt et al, 2013), lvl-CreERT2 (Lapouge et al. 2011) and R26-tdTomato (Madisen et al, 2010) mice were obtained from the Jackson Laboratory. K10-rtTA(Muroyama et al, 2017) mice were obtained from T. Lechler (Duke University), pTRE-H2BGFP (Tumbar et al, 2004) mice were obtained from E. Fuchs (Rockefeller University), Lifeact-GFP (Riedl et al, 2010) mice were obtained from R. Weigert (NIDCR, NIH), GFPNMMIIB (Bao et al, 2007) mice were obtained from R. Adelstein (NHLBI, NIH) and R26p-Fucci2 (Abe et al, 2012) mice were obtained from S.Aizawa (RIKEN). K14-H2BmCherry(Mesa et al, 2015) mice were generated in the laboratory and described previously. All mice used in this study were between 6 and 10 weeks old and were maintained either on a CD1 background (intra-vital imaging) or C57BL/6J. background (scRNA-seq). Mice used for intra-vital imaging were housed on ventilated Tecniplast Iixit racks with ambient temperature of 22 °C and 50% ± 10% humidity with a 12 h:12 h light:dark cycle (07:00–19:00 light). Mice used for scRNA-seq were housed in individually ventilated cages (Tecniplast GM500, Greenline) with controlled temperature and humidity, with a 12h:12h light:dark cycle (06:00-18:00 light).
Wild animals	This study did not involve wild animals.
Reporting on sex	Mice from experimental and control groups were randomly selected from either sex for live imaging experiments. For the newly generated scRNA-seq, female mice were selected to avoid wounds from fighting in the cage
Field-collected samples	This study did not involve field-collected samples.
Ethics oversight	All procedures involving animal subjects were performed under the approval of the Institutional Animal Care and Use Committee (IACUC) of the Yale School of Medicine or the Linköping Animal Ethics Committee in accordance with Swedish Legislation.

Note that full information on the approval of the study protocol must also be provided in the manuscript.

Flow Cytometry

Plots

Confirm that:

☒ The axis labels state the marker and fluorochrome used (e.g. CD4-FITC).

☒ The axis scales are clearly visible. Include numbers along axes only for bottom left plot of group (a 'group' is an analysis of identical markers).

☒ All plots are contour plots with outliers or pseudocolor plots.

☐ A numerical value for number of cells or percentage (with statistics) is provided.

Methodology

Sample preparation	Epidermal cells were isolated from dorsal skin of 8-week old mice as described previously (Joost 2016, DOI: 10.1016/j.cels.2016.08.010). Cells were stained for 1h with fluorophore-conjugated primary antibodies and Sytox blue was added prior to sorting (2 min).
Instrument	FACSAria III (BD Biosciences)
Software	BD FACSDiva 8.0.1 software (BD Biosciences) for sorting and FlowJo (v10, BD Biosciences) for visualizing the gating strategy
Cell population abundance	Final sorted populations made up approximately 1-2% (Tomato+) or 20% (Tomato-) of all cells. Cell type specificity was confirmed after scRNA-sequencing and analysis of the sorted populations.

Gating strategy

Cells were gated as follows: live cells (Sytox-blue/FSC-A), cell size (SSC-A/FSC-A), singlets (FSC-H/FSC-A and SSC-H/SSC-A), basal interfollicular epidermis cells with ITGA6+ (CD49f), LY6A+ (SCA-1) and CD34-, and tracing as Tomato+ and Tomato- cells. Positive and negative gates were assigned based on non-labelled cells. Compensation was applied based on single-stained controls.

☒ Tick this box to confirm that a figure exemplifying the gating strategy is provided in the Supplementary Information.
